# Toxic AGE (TAGE) Theory for the Pathophysiology of the Onset/Progression of NAFLD and ALD

**DOI:** 10.3390/nu9060634

**Published:** 2017-06-20

**Authors:** Masayoshi Takeuchi, Jun-ichi Takino, Akiko Sakasai-Sakai, Takanobu Takata, Mikihiro Tsutsumi

**Affiliations:** 1 Department of Advanced Medicine, Medical Research Institute, Kanazawa Medical University, 1-1 Daigaku, Uchinada, Kahoku, Ishikawa 920-0293, Japan; asakasai@kanazawa-med.ac.jp (A.S.-S.); takajjjj@kanazawa-med.ac.jp (T.T.); 2 Department of Biochemistry, Faculty of Pharmaceutical Sciences, Hiroshima International University, 5-1-1, Hirokoshingai, Kure, Hiroshima 737-0112, Japan; j-takino@ps.hirokoku-u.ac.jp; 3 Department of Hepatology, Kanazawa Medical University, 1-1 Daigaku, Uchinada, Kahoku, Ishikawa 920-0293, Japan; tsutsumi@kanazawa-med.ac.jp

**Keywords:** advanced glycation end-products (AGEs), glyceraldehyde-derived AGEs (GA-AGEs), acetaldehyde-derived AGEs (AA-AGEs), high-fructose corn syrup (HFCS), dietary AGEs, sugar-sweetened beverages (SSB), alcohol beverages

## Abstract

Non-alcoholic fatty liver disease (NAFLD) and alcoholic liver disease (ALD) are among the most common causes of chronic liver diseases in the westernized world. NAFLD and ALD are frequently accompanied by extrahepatic complications, including hepatocellular carcinoma and cardiovascular diseases, which have a negative impact on patient survival. The chronic ingestion of an excessive daily diet containing sugar/high-fructose corn syrup increases the level of the fructose/glucose metabolite, glyceraldehyde (GA), while the chronic consumption of an excessive number of alcoholic beverages increases the level of the alcohol metabolite, acetaldehyde (AA) in the liver. GA and AA are known to react non-enzymatically with the ε- or α-amino groups of proteins, thereby generating advanced glycation end-products (AGEs, GA-AGEs, and AA-AGEs, respectively) in vivo. The interaction between GA-AGEs and the receptor for AGEs (RAGE) alters intracellular signaling, gene expression, and the release of pro-inflammatory molecules and also elicits the production of reactive oxygen species by human hepatocytes and hepatic stellate cells, all of which may contribute to the pathological changes associated with chronic liver diseases. We herein discuss the pathophysiological roles of GA-AGEs and AA-AGEs (toxic AGEs, TAGE) and a related novel theory for preventing the onset/progression of NAFLD and ALD.

## 1. Introduction

Non-alcoholic fatty liver disease (NAFLD) and alcoholic liver disease (ALD) are among the most common causes of chronic liver diseases in the westernized world, and now represent a worldwide public health issue [1,2,3,4]. These diseases have similar pathological spectra, ranging from simple hepatic steatosis to steatohepatitis and liver cirrhosis [5]. A high total energy intake has been positively associated with the onset of NAFLD [6], and specific dietary components have been shown to affect the pathogenesis of this disease [7]. Although several factors may contribute to NAFLD, fructose consumption is regarded as a key player in the onset/progression of this disease [8,9,10,11] and has been reported to induce NAFLD in humans [12,13,14] and rodents [15,16,17,18,19]. The increased ingestion of regular soft drinks was recently linked to NAFLD independent of metabolic syndrome (MetS) [20], with NAFLD patients consuming five-fold more carbohydrates from soft drinks than healthy individuals [21]. NAFLD is defined as hepatic steatosis in the absence of significant alcohol intake (an intake of <20 g ethanol/day for women or <30 g ethanol/day for men is acceptable for the diagnosis as NAFLD), not caused by hepatitis B or C virus, autoimmune hepatitis, the use of hepatotoxic drugs or other compounds, or rare genetic forms [22]. NAFLD is currently the most common chronic liver disorder, with an estimated worldwide prevalence of 25% [2,23,24]. Depending on the method of diagnosis, 65–85% of patients with type 2 diabetes mellitus (T2DM) have NAFLD [25,26]. NAFLD is the second most common reason for being on a waiting list for a liver transplant in the United States [27] and the most common cause of hepatocellular carcinoma (HCC) in the USA [28] and United Kingdom [29]. Non-alcoholic steatohepatitis (NASH) is considered to be the hepatic manifestation of MetS and is associated with central obesity, insulin resistance (IR), T2DM, essential hypertension, and dyslipidemia [30,31]. ALD is the leading cause of liver-related morbidity and mortality worldwide and is a major cause of death among adults with prolonged alcohol abuse [3,4,32]. According to the World Health Organization (WHO), 3.3 million deaths occur worldwide every year due to the harmful use of alcohol, which represents 5.9% of all deaths. The risks of the onset and acceleration of the progression of ALD are both increased by the intake of alcoholic beverages [33,34,35]. The types of beverages consumed may also modify the progression of ALD [36]. Furthermore, drinking patterns are a factor that has been associated with ALD. Previous studies demonstrated that daily or near-daily heavy drinking, not episodic or binge drinking, was closely associated with the development of ALD [33,37]. Moreover, alcohol intake outside of mealtimes and the intake of multiple, different beverages were shown to increase the risk of developing ALD [33].

Advanced glycation end-products (AGEs) are formed by the Maillard reaction, a non-enzymatic reaction between reducing sugars (e.g., glucose, fructose, and glyceraldehyde (GA)) or carbonyl compounds (e.g., glyoxal (GO), methylglyoxal (MGO), and acetaldehyde (AA)) and the ε-amino group of lysine residues, guanidino group of arginine residues, or N-terminal α-amino group of proteins [38,39,40,41]. This process begins with the conversion of reversible Schiff base adducts to more stable, covalently bound Amadori rearrangement products. Over the course of days to weeks, these Amadori products undergo further rearrangement reactions to form irreversibly bound moieties known as AGEs. AGEs were originally characterized by their yellow-brown fluorescent color as well as their ability to form cross-links with and between amino groups; however, this term now encompasses a broad range of advanced products of the glycation process, including *N*-(carboxymethyl)lysine (CML), *N*-(carboxyethyl)lysine (CEL), and *N*-(ethyl)lysine (NEL, which has been called an AA-protein adduct), which do not display color or fluorescence and are not cross-linked proteins [38,39,40,41]. Recent studies have suggested that AGEs are formed not only from reducing sugars (i.e., GA, glucose, and fructose), but also from carbonyl compounds (i.e., AA, GO, and MGO) derived from the auto-oxidation of sugars and other metabolic pathways [41,42,43,44]. Evidence to show that GA- and AA-derived AGEs (GA-AGEs and AA-AGEs) play a role in the pathophysiology of various disorders, such as non-alcoholic/alcoholic liver and brain injury [45,46,47,48,49,50,51,52], cardiovascular diseases (CVD) [44,53,54,55,56,57], T2DM and diabetic vascular complications [41,42,43,44,45,58], Alzheimer’s disease (AD) [43,50,59,60,61,62], and cancer [63,64,65,66,67], is increasing. In the present study, we discussed the pathophysiological roles of GA- and AA-AGEs, predominant components of toxic AGEs (TAGE), and a related novel theory for preventing the onset/progression of NAFLD/NASH and ALD.

## 2. Pathway for the Formation of GA- and AA-AGEs in the Liver

**Figure 1:**
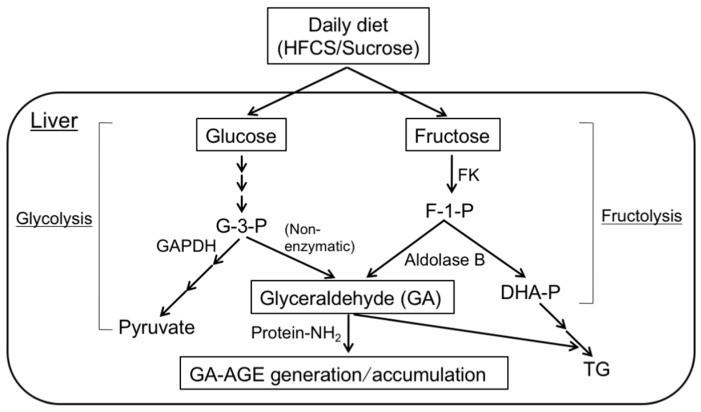
Routes for in vivo GA-AGE generation/accumulation: The chronic consumption of an excessive amount of the daily diet (containing HFCS/sucrose) increases the levels of the sugar metabolite, glyceraldehyde (GA) by fructolysis/glycolysis in the liver. The GA produced induces the generation of GA-AGEs in intracellular compartments. As a result, GA-AGEs accumulate in cells and cause cell damage. HFCS: high-fructose corn syrup; FK: fructokinase; GAPDH: glyceraldehyde-3-phosphate dehydrogenase; G-3-P: glyceraldehyde-3-phosphate; F-1-P: fructose-1-phosphate; DHA-P: dihydroxyacetone-phosphate; TG: triglyceride; GA-AGEs: glyceraldehyde (GA)-derived AGEs; Protein-NH_2_: free amino residues of proteins.

GA is derived from two distinct pathways in the liver: the fructose metabolism pathway (fructolysis) and glycolytic pathway (glycolysis). Fructose is a constituent of high-fructose corn syrup (HFCS) and sucrose, and, hence, is commonly included in the daily diet of humans [68,69]. In fructolysis, fructokinase phosphorylates fructose to fructose-1-phosphate, which is then broken down into GA and dihydroxyacetone-phosphate (DHA-P) by aldolase B [70,71]. The GA produced induces the generation of GA-AGEs in intracellular compartments [43,44,72]. In glycolysis, the enzyme GA-3-phosphate (G-3-P) dehydrogenase (GAPDH) generally breaks down the glycolytic intermediate G-3-P. However, reductions in GAPDH activity lead to the intracellular accumulation of G-3-P. Therefore, G-3-P starts to be metabolized via an alternative pathway, causing increases in the concentration of GA, which, as a result, promotes the generation/accumulation of GA-AGEs [43,44,72] (Figure 1).

**Figure 2:**
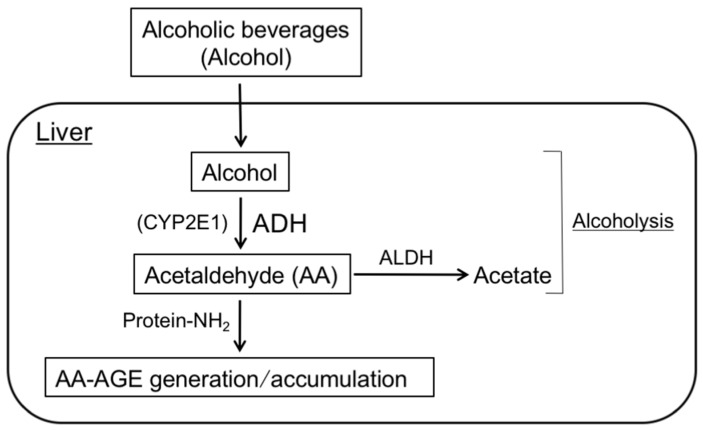
Routes for in vivo AA-AGE generation/accumulation: The chronic consumption of an excessive number of alcoholic beverages increases the levels of the alcohol metabolite, acetaldehyde (AA) by alcoholysis in the liver. The AA produced induces the generation of AA-AGEs in intracellular compartments. As a result, AA-AGEs accumulate in cells and cause cell damage. ADH: alcohol dehydrogenase; ALDH: aldehyde dehydrogenase; CYP2E1: cytochrome P450 family 2, subfamily E, polypeptide 1; AA-AGEs: acetaldehyde (AA)-derived AGEs; Protein-NH_2_: free amino residues of proteins.

AA is derived from the alcohol metabolic pathway (alcoholysis). Alcohol is oxidized in the liver, mainly by alcohol dehydrogenase (ADH), to generate AA, which is, in turn, oxidized by aldehyde dehydrogenase (ALDH) to acetate [73]. Cytochrome P450 family 2, subfamily E, polypeptide 1 (CYP2E1) also contributes to the oxidation of alcohol, but is quantitatively less important than the ADH/ALDH pathway [73]. Liver damage may develop in patients who are abusers of alcohol due to the toxicity of AA, which generates/accumulates AA-AGEs intracellularly (Figure 2).

Due to marked variations in the structures of AGEs found in vivo and the complex nature of the reactions required for their generation, only the structures of some AGEs have been elucidated to date [74]; those of cytotoxic GA- and AA-AGEs currently remain unknown.

## 3. GA-AGE Theory for the Pathophysiology of NAFLD

There is a growing body of evidence to suggest that the interaction between GA-AGEs, but not CML/CEL, and the receptor for AGEs (RAGE) alters intracellular signaling, gene expression, and the release of pro-inflammatory molecules and also elicits the generation of oxidative stress and reactive oxygen species (ROS) in numerous types of cells, all of which may contribute to the pathophysiological changes observed in T2DM, diabetic vascular complications, CVD, AD, cancer, and NAFLD/NASH [42,43,44,45,46,47,48,49,50,61,62,63,64,65,66].

### 3.1. Hepatic IR

Chronic IR results in cellular energy failure, elevated plasma lipids, hypertension, and a predisposition to develop T2DM [75], cerebrovascular diseases and CVD, and cancer [76,77,78,79,80]. IR is now a major public health issue because of its link to obesity, MetS, T2DM, NAFLD/NASH, polycystic ovarian syndrome, and AD.

We previously reported that serum levels of GA-AGEs were elevated under oxidative stress, inflammatory, and/or diabetic conditions and correlated with IR and decreased adiponectin levels, and, thus, is one of the useful biomarkers for differentiating NASH from non-alcoholic fatty liver (NAFL) [46,81,82,83,84,85]. Furthermore, activation of the RAGE downstream pathway by GA-AGEs evokes inflammatory reactions and impairs insulin signaling in human hepatoma Hep3B cells by stimulating the c-Jun N-terminal kinase- and inhibitor-κB kinase-dependent serine phosphorylation of insulin receptor substrate-1 (IRS-1) via Rac-1 activation [86,87,88]. Telmisartan (an angiotensin II type 1 receptor blocker), but not candesartan, blocked GA-AGE signals to hepatic IR, as described above; telmisartan may improve GA-AGE-elicited IR in Hep3B cells by inhibiting the serine phosphorylation of IRS-1, at least in part, via the activation of peroxisome proliferator-activated receptor-γ (PPAR-γ) [87]. Combination therapy with telmisartan and nateglinide (a rapid onset/short duration insulinotropic agent) also improved hepatic IR in Zucker fatty rats by suppressing the GA-AGE-RAGE axis [89]. These findings suggest the involvement of the GA-AGE-RAGE axis in inflammation and IR in the liver.

### 3.2. Cytotoxicity of GA-AGEs in Hepatocytes

Regarding the effects of GA-AGEs on hepatocytes, we previously reported that the GA-AGE-RAGE axis stimulated hepatic C-reactive protein (CRP) in Hep3B cells via the activation of Rac-1 [90]. We found that telmisartan decreased GA-AGE-induced RAGE expression, ROS generation, and subsequent CRP production in Hep3B cells [86]. In addition, GW9662 (an inhibitor of PPAR-γ) blocked the inhibitory effects of telmisartan on RAGE expression and its downstream signaling in Hep3B cells, while troglitazone and ciglitazone, full agonists of PPAR-γ, mimicked the effects of telmisartan on Hep3B cells [86]. These findings suggest that telmisartan blocks GA-AGE signals to CRP expression in Hep3B cells through its anti-oxidative properties via the PPAR-γ-mediated downregulation of RAGE.

RAGE was found to be expressed in the human HCC cell lines, Hep3B, HepG2, and HuH-7, whereas GA-AGEs increased the expression of vascular endothelial growth factor (VEGF) in Hep3B cells [66,91]. Furthermore, GA-AGE-treated conditioned medium significantly increased the proliferation, migration, and tube formation of endothelial cells (ECs), suggesting that GA-AGE-RAGE signaling enhances the angiogenic potential of Hep3B cells by upregulating VEGF expression [66]. GA-AGEs have also been shown to increase the growth of HuH-7. MK615, an extract from Japanese apricot, was previously reported to inhibit the GA-AGE-induced proliferation of HuH-7 by suppressing RAGE expression [91]. An orally administered high glucose-derived AGE (Glu-AGEs) beverage induced hepatic VEGF and RAGE expression as well as GA-AGE accumulation in rats, suggesting a pathophysiological role for dietary Glu-AGEs in the onset/progression of HCC [92].

We previously demonstrated that GA-AGE-RAGE axis-mediated, NADPH oxidase (NOX)-induced ROS generation stimulated the proliferation and tube formation of ECs, key steps in tumor angiogenesis, through VEGF expression via the transcriptional activation of nuclear factor-κB (NF-κB) and activator protein-1 [93,94,95,96,97]. Furthermore, the activation of the GA-AGE-RAGE axis evoked inflammatory and thrombogenic reactions in ECs by inducing plasminogen activator inhibitor-1, intercellular adhesion molecule-1, and monocyte chemoattractant protein-1 (MCP-1) expression via the generation of ROS [44,98,99,100].

### 3.3. Cytotoxicity of GA-AGEs in Hepatic Stellate Cells (HSCs)

HSCs are the main extracellular matrix-producing cells in the liver, and, thus, play a pivotal role in liver fibrogenesis [101]. Fehrenbach et al. showed that HSCs and myofibroblasts (MFB) expressed RAGE, its expression was increased during the activation of HSCs and process of differentiation to MFB, and it was modulated by transforming growth factor-β1 (TGF-β1). The ligand activation of RAGE led to the formation of ROS and induction of mitogen-activated protein kinase and NF-κB signaling pathways in HSCs [101]. We demonstrated that GA-AGEs induced inflammation- and fibrogenesis-related gene and protein expression, such as TGF-β1, collagen-type Iα2, and MCP-1, in the cultured HSC line, LI90 via the NOX-mediated generation of ROS [47].

### 3.4. Cytotoxicity of Intracellular GA-AGEs in Hepatocytes

We previously reported that GA induced concentration- and time-dependent cell death and increased the intracellular concentration of GA-AGEs in Hep3B cells [102]. Aminoguanidine, which inhibits the formation of AGEs, reduced the generation/accumulation of intracellular GA-AGEs and prevented GA-AGE-induced cell death. As a consequence, the formation of intracellular GA-AGEs was associated with cell death. We previously reported that a GA-AGE-modified protein of 70 kDa, which we identified as heat shock cognate 70 (Hsc70), was detected the earliest and in the greatest abundance in GA-treated Hep3B cells [102]. We also found that GA modifications reduced the ability of Hsc70 to refold denatured luciferase activity. Taken together, these findings suggest that the loss-of-function of Hsc70 by glycation induces cell death. We also demonstrated that intracellular GA-AGEs increased the expression of the mRNA of the acute phase reactant CRP [102]. These findings prompted us to speculate that intracellular GA-AGEs play roles in the pathophysiology of NAFLD/NASH.

### 3.5. Intracellular GA-AGE Generation in Fructose

The excessive intake of fructose contributes to the onset of NAFLD and its progression to NASH. Fructose is metabolized to GA by fructokinase and aldolase B in the liver. We showed that intracellular GA-AGEs were generated in the presence of fructose. We also demonstrated that GA-AGEs ameliorated IR in mice fed a high-fat, high-fructose diet [103]. These findings suggest that hepatic GA-AGEs are useful markers for the diagnosis and therapeutic evaluation of IR and may play a pathophysiological role in the onset of IR. Additionally, heterogeneous nuclear ribonucleoprotein M (hnRNPM) was identified as one of the target proteins of GA-AGEs [104]. These findings suggest that GA-AGE-modified hnRNPM, resulting from the exposure of cells to fructose, alters gene expression and exerts adverse effects in Hep3B cells. The generation/accumulation of GA-AGEs results in hepatocyte damage, GA-AGEs leak into the blood, and, thus, GA-AGE levels in circulating fluids are considered to increase.

### 3.6. Serum GA-AGE Levels in NAFL/NASH/HCC

We previously reported that GA-AGEs, but not Glu-AGEs or CML were present in sera at significantly higher concentrations in patients with NASH than in those with NAFL and healthy controls. Furthermore, the immunohistochemical staining of GA-AGEs showed intense staining in the livers of patients with NASH [46,48]. We also found that serum GA-AGE levels may play a role in the pathophysiology of NASH and have potential as a biomarker for discriminating NASH from NAFL on the basis of the following evidence: (i) serum GA-AGE levels positively correlated with the homeostatic model assessment of IR and were inversely associated with adiponectin levels; (ii) although serum GA-AGE levels did not correlate with the severity of hepatic steatosis or fibrosis, they were not affected by the status of glucose tolerance.

Atorvastatin decreased the serum levels of GA-AGEs in NASH patients with dyslipidemia [85]. Serum levels of GA-AGEs have been correlated with those of alanine aminotransferase, tumor necrosis factor-α (TNF-α), type IV collagen 7S, and procollagen type III propeptide [85]. These beneficial effects were attributed to the anti-inflammatory activity of atorvastatin and its downregulation of the hepatic expression of fructokinase, which inhibits fructose metabolism in the liver [105]. These findings suggest that GA-AGEs play critical roles in the pathophysiology of NASH and may serve as potential targets for therapeutic interventions [106,107,108,109].

We very recently reported that circulating GA-AGE levels were significantly higher in non-B or non-C (NBNC)-HCC patients than in NASH subjects without HCC or control subjects [110]. In a multiple stepwise regression analysis, age, γ-glutamyl transpeptidase, and high-density lipoprotein cholesterol (inversely) remained significant and were independently related to GA-AGE levels. These findings suggest that GA-AGEs are involved in the pathophysiology of NBNC-HCC, and, thus, have potential as biomarkers with the ability to discriminate NBNC-HCC from NASH.

### 3.7. Serum GA-AGE Levels in CVD

Previous studies highlighted the relationship between NAFLD and CVD [111,112,113]. The most important characteristic of this relationship is increased mortality and morbidity. Mortality in NAFLD patients is higher than that in the general population and is mainly defined by concomitant CVD and hepatic dysfunction.

In recent studies, we found that: (i) GA-AGE levels, but not those of hemoglobin A1c (HbA1c) or CML were independently associated with vascular inflammation, as evaluated by [^18^F] fluorodeoxyglucose positron emission tomography in outpatients [114]; (ii) GA-AGE levels were one of the independent correlates of the decreased number and impaired migratory activity of circulating endothelial progenitor cells in apparently healthy subjects [115], suggesting the involvement of GA-AGEs in impaired EC repair; (iii) high baseline GA-AGE levels were associated with plaque progression in an assessment of pitavastatin and atorvastatin in an acute coronary syndrome trial (the JAPAN-ACS Sub-study) in Japan [116]; and (iv) GA-AGE levels were significantly higher in the high mean amplitude of glycemic excursions (MAGE) group than in the low MAGE group in pre-DM patients [117]. These findings indicate that serum GA-AGE levels have potential as a biomarker for predicting the progression of atherosclerosis and future cardiovascular events.

### 3.8. Neurotoxicity of GA-AGEs

Growing evidence supports the concept that AD is fundamentally a metabolic disease with molecular and biochemical features that correspond to T2DM and other peripheral IR disorders. Brain IR and its consequences may readily account for most of the structural and functional abnormalities associated with AD. However, disease pathophysiology is complicated by AD occurring as a separate disease process or arising in association with systemic IR diseases, including obesity, T2DM, and NAFLD [118].

We previously demonstrated that GA-AGEs were strongly neurotoxic in cortical neuronal cells [50,59]. The neurotoxicities of GA-AGEs were stronger than those of Glu-AGEs and CML, two extensively examined AGE species. Moreover, the neurotoxic effects of serum AGE fractions from diabetic nephropathy in hemodialysis patients were completely attenuated by the addition of an anti-GA-AGE antibody, but not the antibodies of other AGEs [50,59]. In AD brains, GA-AGEs were mainly detected in the cytosol of neurons in the hippocampus and parahippocampal gyrus, but not in senile plaques or astrocytes [60]. These findings suggest that the generation of GA-AGEs during sugar metabolism may not only be cytotoxic to hepatocytes, but also to neuronal cells and possibly many other cells, thereby inducing cellular and organ impairments.

Evidence obtained over the past 20 years has indicated that amyloid β_1–42_ (Aβ42) levels in the cerebrospinal fluid (CSF) of AD patients are significantly lower than those in age-matched healthy elderly controls, whereas total tau and p-tauT181 levels are significantly higher [119]. Furthermore, the levels of other AD biomarkers, such as VEGF [120] and TGF-β1 [121], were found to be higher in the CSF of AD patients. We recently demonstrated that intracellular GA-AGE generation decreased Aβ42 levels and increased total tau and p-tauT181 levels in culture media and also increased the intracellular levels of total tau, p-tauT181, VEGF, and TGF-β in human neuroblastoma SH-SY5Y cells [122]. Although the exact mechanisms underlying the targets of GA-AGEs and its downstream signaling pathway currently remain unclear, the measurement of GA-AGE levels in the CSF and/or serum may be a useful biomarker for the early detection of AD [123,124].

## 4. AA-AGE Theory for the Pathogenesis of ALD

The pathophysiology of ALD is a dynamic process that is triggered by complex interactions between the metabolic intermediates of alcohol, inflammation, and immune responses from cellular injury [125,126]. Since hepatocytes are the primary site of alcohol detoxification, its major toxic metabolic intermediate, AA causes direct hepatocyte damage and also generates/accumulates AA-AGEs [127].

### 4.1. Cytotoxicity of AA-AGEs in Hepatocytes

We recently demonstrated that AA-AGEs induced hepatotoxicity and hepatocyte death in rat primary hepatocyte cultures, whereas NEL did not. The hepatotoxicity of AA-AGEs was neutralized by the addition of an anti-AA-AGE antibody, but not by an anti-NEL antibody, suggesting that AA-AGEs are also toxic to hepatocyte cells [52]. The NEL (AA-protein adduct) pathway for the reaction of Amadori compounds may be a physiologically relevant mechanism for averting the production of AA-AGEs, and, thus, preventing potential cellular toxicity arising from AA-AGE formation during alcoholism.

### 4.2. Cytotoxicity of AA-AGEs in HSCs

We demonstrated that AA-AGEs induced oxidative stress and generated ROS in rat HSCs [52]. Untreated control HSCs showed mild staining for 4-hydroxy-2-nonenal (4-HNE), an oxidative stress marker. A treatment with AA-AGEs produced marked increases in the staining intensity of 4-HNE. More than 90% of cultured HSCs treated with AA-AGEs were viable. A few apoptotic cells were present in cultures treated with AA-AGEs. A significant increase was observed in the staining intensity of 4-HNE in HSCs treated with AA-AGEs. These findings suggest that AA-AGEs bind to AGE receptors (ex. RAGE), induce oxidative stress, and generate ROS, which then trigger steatosis, hepatocyte ballooning, and the pathophysiology of ALD. However, the exact molecular mechanisms underlying the induction of oxidative stress and generation of ROS remain unclear.

### 4.3. Hepatic AA-AGEs Reflect the Degree of ALD during the Chronic Consumption of Alcohol

We found a marked increase in staining for AA-AGEs in the pericentral areas of rat livers treated with alcohol. Staining intensity significantly increased from four to eight weeks, indicating that the generation/accumulation of AA-AGEs during the chronic consumption of alcohol was additive and also in parallel with the intensity of hepatic fatty degeneration and ALD. Furthermore, we observed the intense staining of AA-AGEs and 4-HNE surrounding the central vein during the pathophysiology of ALD as well as during the early abstinence period [52].

AA-AGEs that were generated or accumulated during the chronic consumption of alcohol were eliminated with abstinence. The intensity of staining for AA-AGEs markedly increased in the hepatocytes of the perivenular region until week 8 during abstinence, but was significantly reduced by 10 weeks and completely disappeared after 12 weeks. Steatosis also simultaneously and completely disappeared after 12 weeks with the restoration of the lobular architecture of hepatic tissue [52]. These findings prompted us to speculate that intracellular AA-AGEs play roles in the pathophysiology of ALD.

### 4.4. Staining of AA-AGEs in ALD Patients

We obtained representative staining images for steatosis, AA-AGEs, and 4-HNE in ALD patients and control subjects [52]. AA-AGE and 4-HNE staining was completely absent in control livers. A histopathological evaluation demonstrated severe fatty degeneration in all ALD patients examined. There was strong staining for AA-AGEs and 4-HNE in the entire tissue specimen, and the staining pattern and its intensity strongly correlated, indicating an underlying mechanism for AA-AGE generation/accumulation and ROS generation or vice versa. These findings indicate that AA-AGEs induce the generation of ROS and contribute to the pathophysiology of ALD. Our previous findings further support this theory [51].

### 4.5. Neurotoxicity of AA-AGEs

We found that the incubation of cortical neurons with AA-AGEs generated a dose-dependent increase in neuronal cell death, and the neurotoxicity of AA-AGEs was neutralized by the addition of an anti-AA-AGE antibody, but not by an anti-NEL antibody, suggesting that AA-AGEs are also toxic to neuronal cells [51]. *N*-acetylcysteine, an antioxidant, prevented AA-AGE-induced neuronal cell death. Therefore, ROS generation induced by AA-AGEs may be involved in neuronal cell death. We also detected the AA-AGE epitope in the brains of patients with alcoholism. Alcoholic brain homogenates revealed immunoreactivity with an apparent molecular weight of 185 kD. Upadhya et al. reported that chronic ethanol-treated rat brains immunostained with an anti-NEL antibody showed bands with approximate molecular masses of 188, 107, 60, and 50 kD [128]; our findings indicate that the AA-AGE structure plays an important role in the neuropathological processes associated with human alcoholism. These findings also suggest that the generation/accumulation of AA-AGEs during alcoholism is not only toxic to hepatocytes, but also to neuronal cells and potentially many other cells, thereby inducing cellular and organ impairments.

## 5. Prevention of the Generation/Accumulation of GA- and AA-AGEs in the Liver

Previous studies reported that the chronic ingestion of excessive amounts of sugar (typically HFCS/sucrose)-containing beverages/foods not only caused obesity, MetS, and T2DM, but was also involved in the onset/progression of NAFLD/NASH, CDV, and AD; however, the underlying mechanisms currently remain unknown [129,130,131,132,133]. We revealed that GA-AGEs strongly correlated with lifestyle-related diseases (LSRD) [134]. The chronic ingestion of excessive amounts of SSB, which contain HFCS/sucrose and dietary AGEs (particularly Glu-AGEs), increased the levels of the fructose/glucose metabolite, GA in the liver.

### 5.1. Sugars (HFCS/Sucrose)

The sugar additives found in many SSB and commercial products are widely viewed as the main source of the increased amounts of fructose consumed in developed countries. Glucose is a major energy source for the entire body, while fructose metabolism mainly occurs in the liver. Fructose may be a key contributor to the biochemical alterations that promote MetS, T2DM, and NAFLD [135,136,137,138,139]. Alwahsh and Gebhardt reviewed the physiological influences of fructose consumption that lead to NAFLD and its associated abnormalities, such as the MetS, IR, and disruption of the gut microbiota. Furthermore, they suggested a novel hypothesis for how fructose may affect the liver and reviewed possible therapeutic options for fructose-induced NAFLD [140].

#### 5.1.1. GA-AGE Generation/Accumulation

A growing body of epidemiological and mechanistic evidence argues that excessive sugar consumption affects human health beyond the simple addition of calories [141]. Sugar has been implicated in the onset of all diseases associated with MetS [142,143], including hypertension, CVD, NAFLD/NASH, T2DM, and the aging process, which is promoted by damage to proteins due to the non-enzymatic binding of sugars (so-called glycation) [38,39,40,41]. We recently reported that the fructose- and glucose-induced generation of GA caused GA-AGE generation/accumulation, which may be used as biomarkers to predict LSRD [134].

#### 5.1.2. Sugar Content in Soft Drinks and Alcoholic Beverages

Fast-food meals, which are characterized by standardized palatability, high contents of fat and sugar, large portion sizes, and high energy densities, are frequently consumed with alcoholic beverages. Alwahsh et al. provided evidence for the synergistic effects of fructose and alcohol with a high-fat-diet on dyslipidemia and IR-accompanied liver damage in a rat model [144]. In the setting of a pandemic of obesity and T2DM, the American Heart Association (AHA) recently released scientific recommendations to reduce added sugar intake to no more than 25 g (women)-37.5 g (men) sugar/day for most Americans [145]. A new WHO guideline in 2015 recommended that adults and children reduce their daily intake of added sugars to less than ca. 25 g sugar/day, which may provide additional health benefits [146]. We recently reported the amounts of total sugar in typical soft drinks (750 kinds) in Japan. Approximately 43% of the soft drinks tested contained 25 g or more sugar per bottle/can based on standard serving sizes [147].

Alcoholic beverages based on Japan’s liquor tax law are classified into four types: (i) effervescent alcoholic beverages (including beer and low-malt beer); (ii) brewed alcoholic beverages (such as sake and fruit wine); (iii) distilled alcoholic beverages (including distilled spirits, whisky, brandy, and spirits); and (iv) mixed liquor (such as liqueurs and sweet fruit wine). The average sugar content and number of alcoholic beverages in each classification of total sugars are shown in Table 1. Approximately 24% of the alcoholic beverages (45% of mixed liquor) tested contained 25 g (AHA/WHO guidelines) or more sugar per bottle/can/glass based on standard serving sizes (30–350 mL).

#### 5.1.3. Restricting the Consumption of SSB and Alcoholic Beverages

Imamura et al. prospectively examined the relationship between the consumption of SSB and risk of T2DM from 17 cohort studies [148]. They repeated a meta-analysis to estimate the relative risk for 250 mL/day. A 350-mL bottle/can of mixed liquor contains approximately 20–40 g of added sugars; therefore, the consumption of one bottle/can equals the recommended amount of added sugars for one day [147].

The findings of our studies suggest that sugars are present at appreciable levels in soft drinks and alcoholic beverages, and may contribute to the accumulation of GA-AGEs in the body. The contents of HFCS/sucrose in soft drinks, foods, and alcoholic beverages need to be taken into consideration for disease prevention, particularly in individuals at high risk of the onset of NAFLD/NASH. These findings are promising in that the concept of the restricted intake of sugars in soft drinks, foods, and alcoholic beverages (particularly mixed liquor) may be a new strategy when considering the suppressed generation/accumulation of GA-AGEs and prevention of NAFLD/NASH.

### 5.2. Dietary Glu-AGEs

The fructose/glucose metabolite, GA is known to react non-enzymatically with the ε- and α-amino groups of proteins in order to generate GA-AGEs, enhance the generation/accumulation of GA-AGEs, upregulate RAGE mRNA levels, and increase serum GA-AGE levels, leading to GA-AGE-RAGE interactions. We previously reported increases in hepatic RAGE expression and the enhanced generation/accumulation of GA-AGEs after the oral consumption of Glu-AGE-rich beverages by normal rats [92]. SSB, which contain large amounts of sugars [147] and/or dietary AGEs [149], need to be taken into consideration for disease prevention, particularly in individuals at high risk of the onset of LSRD.

#### 5.2.1. GA-AGE Generation/Accumulation

Two major sources of AGEs, exogenous and endogenous AGEs, have been identified in humans [150,151,152,153,154]. We recently indicated that serum levels of GA-AGEs, but not those of HbA1c, Glu-AGEs, or CML have potential as biomarkers to predict the progression of LSRD [134]. We also demonstrated an increase in the expression of hepatic RAGE and VEGF, and the enhanced generation/accumulation of GA-AGEs in normal rats administered Glu-AGE-rich beverages that did not contain GA-AGEs [92,149]. These findings indicate that Glu-AGEs, which are normally contained in beverages/foods [149] and are taken orally into the body, enhance the generation/accumulation of GA-AGEs and increase serum GA-AGE levels, leading to GA-AGE-RAGE interactions [155].

#### 5.2.2. Glu-AGE Contents in Soft Drinks, Foods, and Alcoholic Beverages

Dietary consumption has recently been identified as a major environmental source of pro-inflammatory AGEs in humans. It is currently being disputed whether dietary AGEs represent a risk to human health [156,157,158]. We evaluated the amounts of AGEs and sugars in beverages and foods, which cause the generation/accumulation of GA-AGEs in the body.

We evaluated the amounts of four kinds of AGEs (Glu-AGEs, fructose-derived AGEs (Fru-AGEs), CML, and GA-AGEs) in 750 kinds of soft drinks and 767 kinds of foods using a competitive ELISA involving specific antibodies [159,160,161,162]. Glu-AGEs and Fru-AGEs (particularly Glu-AGEs), but not CML or GA-AGEs were present at appreciable levels in beverages and foods commonly consumed in Japan. Glu-AGEs were present at significant concentrations in ≥85% of the tested beverages and in ≥82% of the tested foods [149].

Furthermore, Glu-AGEs and Fru-AGEs (particularly Glu-AGEs), but not CML or GA-AGEs were present at appreciable levels in 135 alcoholic beverages commonly consumed in Japan. Glu-AGEs were present at significant concentrations in ≥90% of the alcoholic beverages tested [149]. The average Glu-AGE content and number of alcoholic beverages in each classification of Glu-AGEs are shown in Table 2. The amount of Glu-AGEs was more than 20,000 U/bottle in ca. 40% of the alcoholic beverages examined.

#### 5.2.3. Restricting the Consumption of Glu-AGEs

In humans, approximately 10% of AGEs (estimated CML) in beverages and foods are taken into the body. Of these, ~1/3 are excreted in urine within 48 h of their consumption, while ~2/3 accumulate within the body [163]. We examined blood Glu-AGE levels in mostly healthy subjects and found that they were 10–20 U/mL. The dietary intake of beverages/food products containing large amounts of Glu-AGEs has been shown to have negligible effects on the body because it may result in elevated concentrations of Glu-AGEs and sugar in the blood and promote the hepatic generation/accumulation of GA-AGEs [92,155]. Kremezin, an oral adsorbent that slows the onset of chronic renal failure (CRF) by promoting the removal of uremic toxins, was found to reduce blood Glu-AGE and GA-AGE levels in non-T2DM CRF patients [155].

There is accumulating evidence to show the pathological role of dietary AGEs in various cardiometabolic disorders and aging [164,165]. These findings are promising because the concept of the restricted intake of Glu-AGEs in soft drinks/foods and alcoholic beverages (particularly mixed liquor) may be a new strategy when considering the suppressed generation/accumulation of GA-AGEs and prevention of NAFLD/NASH.

## 6. Conclusions and Perspectives

**Figure 3:**
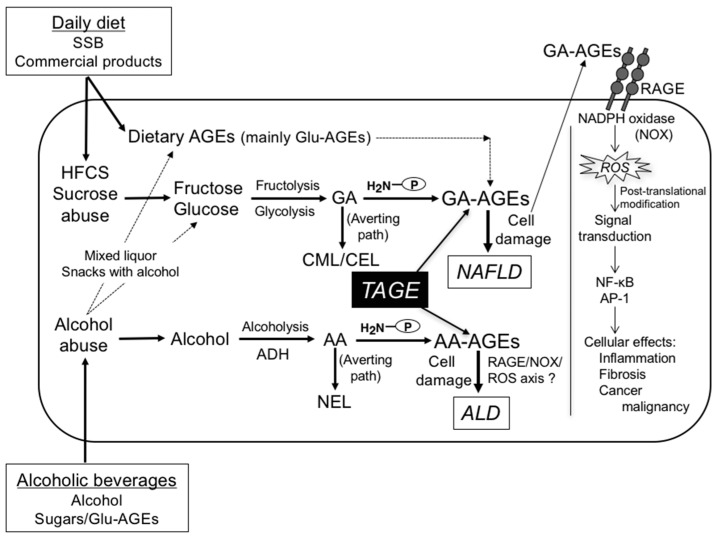
The toxic AGE (TAGE) theory for the pathophysiology of NAFLD and ALD: The chronic ingestion of an excessive daily diet (SSB and commercial products, which contain HFCS/sucrose) increases the levels of the sugar metabolite, glyceraldehyde (GA), while the chronic consumption of an excessive number of alcoholic beverages increases the levels of the alcohol metabolite, acetaldehyde (AA) in the liver. GA or AA is known to react non-enzymatically with the ε- or α-amino groups of proteins to form reversible Schiff bases and then Amadori products. These early glycation products undergo further complex reactions such as rearrangement, dehydration, and condensation to become irreversibly cross-linked, heterogeneous fluorescent derivatives termed “AGEs” (GA-AGEs or AA-AGEs). The CML/CEL or NEL pathway for the reaction of Amadori products may be a physiologically relevant mechanism for averting the generation of GA-AGEs or AA-AGEs (the predominant components of TAGE), and, thus, prevent potential cellular toxicity arising from the generation of TAGE in vivo. Furthermore, the chronic ingestion of excessive dietary AGEs (mainly Glu-AGEs) further promotes the enhanced generation/accumulation of GA-AGEs and expression of RAGE. As a result, GA-AGEs accumulate in cells, cause cell damage, and leak into the blood, and, thus, GA-AGE levels in circulating fluids may be considered to increase. Extracellular GA-AGEs induce inflammation, fibrosis, and cancer malignancy via RAGE-NOX-ROS signaling. The GA-AGE-RAGE axis may lead to ROS generation by NOX. Oxidative hepatic injury may result from the direct attack of ROS on essential biomolecules, including lipids, proteins, and DNA, with the subsequent activation of cell death pathways and loss of biological functions and hepatocyte viability. ROS may indirectly activate redox sensitive transcription factors, including NF-κB and activator protein-1 (AP-1), which trigger the production of cytotoxic, pro-inflammatory, and fibrogenic mediators by HSCs, thereby promoting NAFLD disease progression. AA-AGEs may also bind to RAGE and generate ROS, which trigger signal transduction in hepatocytes/HSCs. However, the exact molecular mechanisms underlying the generation of ROS by AA-AGEs remain unclear. Alcoholic beverages (particularly mixed liquor) contain large amounts of sugars and Glu-AGEs may generate/accumulate TAGE (AA-AGEs and GA-AGEs). Taken together, our theory suggests that TAGE are novel therapeutic targets for preventing lifestyle-related diseases. Therefore, inhibiting the generation/accumulation of TAGE is a promising target for the novel prevention of and therapeutic interventions for NAFLD and ALD. SSB: sugar-sweetened beverages; HFCS: high-fructose corn syrup; ADH: alcohol dehydrogenase; GA: glyceraldehyde; GA-AGEs, GA-derived AGEs; AA: acetaldehyde; AA-AGEs, AA-derived AGEs; CML, *N*-(carboxymethyl)lysine; CEL, *N*-(carboxyethyl)lysine; NEL, *N*-(ethyl)lysine; NAFLD, non-alcoholic fatty liver disease; ALD, alcoholic liver disease; TAGE, toxic AGEs; RAGE: receptor for AGEs; ROS: reactive oxygen species; P-NH_2_, free amino residues of proteins.

NAFLD and ALD are generally related to unhealthy lifestyle habits, including the excessive daily intake of SSB/commercial products and alcohol beverages, and both are likely to become serious health issues in the future. In contrast to chronic viral liver diseases, NAFLD and ALD are frequently accompanied by extrahepatic diseases that may influence patient survival. We demonstrated that GA-AGEs may play a role in the pathophysiology of NAFLD/NASH in humans [46,48,85,106,107]. Serum levels of GA-AGEs, but not HbA1c, Glu-AGEs, or CML, were significantly higher in NASH patients than in those with NAFL and healthy controls [46,48,108]. As a result, GA-AGEs accumulate in cells, cause cell damage, and leak into the blood; therefore, GA-AGE levels in circulating fluids may be considered to increase (Figure 3). Interactions between GA-AGEs and RAGE alter intracellular signaling, gene expression, and the release of pro-inflammatory molecules, all of which may contribute to the pathophysiological changes observed in NAFLD/NASH. Although the generation of ROS has been observed in numerous types of cells (i.e., hepatocytes and neuronal cells) in NAFLD/NASH, it remains controversial whether ROS themselves contribute to the development of NAFLD/NASH. ROS are essential in the physiologically normal regulation of subcellular bioenergy systems, proteolysis regulation, transcription activation, enzyme activation, mitochondrial DNA changes, and the redox regulation of metabolism and cell differentiation [166]. We also demonstrated that AA-AGEs were toxic to hepatocytes and neuronal cells, whereas NEL was not. The chronic consumption of alcohol generated AA-AGEs in pericentral hepatocytes from the metabolic products of alcohol, mainly AA, and induced oxidative stress, leading to the production of ROS in a rat model. The staining of AA-AGEs correlated with the severity of ALD in rats and humans. The chronic consumption of alcohol has been shown to generate/accumulate AA-AGEs, which contribute to the pathophysiology of ALD [52]. Alcoholic beverages (particularly mixed liquor) contain alcohol, sugar/HFCS, and Glu-AGEs; therefore, they generate/accumulate not only AA-AGEs, but also GA-AGEs (Figure 3). The characteristics of modern dietary habits (excessive intake of sugar/HFCS, Glu-AGEs-rich SD/foods, and alcoholic beverages) promote the generation/accumulation of GA-AGEs and AA-AGEs in the liver, and are strongly involved in the onset/progression of NAFLD and ALD. Thus, our findings provide a new concept for preventative measures against NAFLD and ALD.

In conclusion, of the various types of AGE structures generated in vivo, those of TAGE (GA-AGEs and AA-AGEs), but not non-TAGE (including CML/CEL and NEL) may play an important role in the pathophysiological processes associated with the generation/accumulation of TAGE (Figure 3) [41,42,43,44,52,106,107,108,109,134]. Additional clinical investigations may provide us with more information on whether the restriction of dietary sugars/Glu-AGEs and alcoholic beverages is beneficial for preventing the onset/progression of LSRD and represents a novel therapeutic target to prevent NAFLD and ALD.

## Figures and Tables

**Table 1:** Number of alcoholic beverages tested for sugar content.

	(g per Bottle/Can/Glass)				
	Sugar Content		≥25	12.5–24.9	<12.5
	(Average)	(Min–Max)			
Alcoholic beverages (135)	18.5				
Effervescent alcoholic beverages (21)	16.4	(5.6–23.8)		14	7
Brewed alcoholic beverages (27)	12.2	(6.6–24.1)		11	16
Distilled alcoholic beverages (16)	7.0	(2.9–12.3)			16
Mixed liquor (71)	24.1	(5.0–43.8)	32	34	5
(Number of alcoholic beverages)			(32)	(59)	(44)

**Table 2:** Number of alcoholic beverages tested for Glu-AGE content.

	(U per Bottle/Can/Glass)				
	Glu-AGE Content		≥50,000	20,000–49,999	<20,000
	(Average)	(Min–Max)			
Alcoholic beverages (135):	17,750				
Effervescent alcoholic beverages (21)	1010	(0–9210)			21
Brewed alcoholic beverages (27)	2600	(0–23,290)		1	26
Distilled alcoholic beverages (16)	9430	(0–26,210)		4	12
Mixed liquor (71)	30,340	(0–64,710)	6	45	20
(Number of alcoholic beverages)			(6)	(50)	(79)

## Abbreviations

The following abbreviations are used in this manuscript:

AA	Acetaldehyde
AA-AGEs	Acetaldehyde-derived AGEs
Aβ	Amyloid beta
AD	Alzheimer’s disease
ADH	Alcohol dehydrogenase
AGEs	Advanced glycation end-products
AHA	American Heart Association
ALD	Alcoholic liver disease
ALDH	Aldehyde dehydrogenase
CEL	*N*-(Carboxyethyl)lysine
CML	*N*-(Carboxymethyl)lysine
CRF	Chronic renal failure
CRP	C-Reactive protein
CSF	Cerebrospinal fluid
CVD	Cardiovascular disease
CYP2E1	Cytochrome P450 family 2, subfamily E, polypeptide 1
ECs	Endothelial cells
ELISA	Enzyme-linked immunosorbent assay
Fru-AGEs	Fructose-derived AGEs
GA	Glyceraldehyde
GA-AGEs	Glyceraldehyde-derived AGEs
GA-3-P	Glyceraldehyde-3-phosphate
GAPDH	Glyceraldehyde-3-phosphate dehydrogenase
Glu-AGEs	Glucose-derived AGEs
HbA1c	Hemoglobin A1c
HCC	Hepatocellular carcinoma
HFCS	High-fructose corn syrup
4-HNE	4-Hydroxy-2-nonenal
hnRNPM	Heterogeneous nuclear ribonucleoprotein M
Hsc70	Heat shock cognate 70
HSCs	Hepatic stellate cells
IR	Insulin resistance
IRS-1	Insulin receptor substrate-1
LSRD	Lifestyle-related diseases
MCP-1	Monocyte chemoattractant protein-1
MetS	Metabolic syndrome
MFB	Myofibroblasts
NAFL	Non-alcoholic fatty liver
NAFLD	Non-alcoholic fatty liver disease
NASH	Non-alcoholic steatohepatitis
NBNC-HCC	Non-B or non-C HCC
NEL	*N*-(Ethyl)lysine
NF-κB	Nuclear factor-κB
PPAR-γ	Peroxisome proliferator-activated receptor-γ
RAGE	Receptor for AGEs
ROS	Reactive oxygen species
SSB	Sugar-sweetened beverages
TAGE	Toxic AGEs
T2DM	Type 2 diabetes mellitus
TGF-β1	Transforming growth factor-β1
TNF-α	Tumor necrosis factor-α
VEGF	Vascular endothelial growth factor
WHO	World Health Organization
